# Prognostic factors for stereopsis in refractive accommodative esotropia

**DOI:** 10.12669/pjms.314.7465

**Published:** 2015

**Authors:** Hande Guclu, Vuslat Pelitli Gurlu, Sadik Altan Ozal, Zeynep Gursel Ozkurt

**Affiliations:** 1 Hande Guclu, MD Ophthalmologist, Trakya University of Medicine, Ophthalmology Department, Edirne, 22030, Turkey; 2 Vuslat Pelitli Gurlu, MD Associate Professor, Trakya University of Medicine, Ophthalmology Department, Edirne, 22030, Turkey; 3 Sadik Altan Ozal, MD Ophthalmologist, Trakya University of Medicine, Ophthalmology Department, Edirne, 22030, Turkey; 4 Zeynep Gursel Ozkurt, MD Assistant Professor, Ophthalmology Department, Dicle University of Medicine, Diyarbakir, 21070, Turkey

**Keywords:** Amblyopia, Refractive accommodative esotropia, Stereopsis

## Abstract

**Objective:**

To determine the prognostic factors affecting stereoacuity in patients with refractive accommodative esotropia (RAE) according to the results of long follow- up period.

**Methods:**

We reviewed the charts of 70 patients with RAE between the years 1985-2014. Patients were classified into three groups. G-1: Stereoacuity score 40 second/arc. G-2: Stereoacuity score >40 second/arc (50-3000). G-3: No binocular vision. Initiation age of RAE, duration of deviation, refractive error, amblyopia, amblyopia treatment, anisometropia, visual acuity, family history, angle of deviation for distance and near at each group and the prognostic factors affecting stereoacuity were analyzed.

**Results:**

The mean initiation age of RAE was 2.7±1.5 years, the mean age at first visit was 6.4±4.2 years. The mean follow up time was 7.3±4.4 years. Seven patients had 40 second/arc, 48 patients had 50 to 3000 second/arc stereoacuity, 15 patients had no binocular vision. Mean deviation for near was statistically higher in group 2 and 3. Visual acuity levels were higher in group 1 and 2 and was statistically significant. Low visual acuity (p=0.001, 0.008), higher angle of deviation at near (p=0.01), increased duration of deviation (p=0.01), presence of amblyopia (p=0.001) and irregularity of amblyopia treatment (p=0.01) were significantly related with poor stereoacuity.

**Conclusion:**

According to the prognostic factors low stereoacuity was mostly related with amblyopia as a result the late presentation of the patients in seeking care. Appropriate treatment as full refractive correction and amblyopia treatment during the RAE is important for development of good stereopsis. Also angle of deviation at near and duration of deviation can be a useful predictor for poor stereoacuity levels.

## Introduction

Accommodative esotropia is described as convergent deviation of the eyes related with activation of accommodation reflex. Refractive accommodative esotropia (RAE) includes accommodative convergence, uncorrected hyperopia and inadequate fusional divergence.^1^-^3^ Uncorrected hyperopia coerces patient to accommodate to net retinal image. So, this causes increased convergence. Esotropia will appear if fusional divergence of the patient is inadequate to compensate for increased convergence.^4^ Patients treated by prescription of full correction of hyperopia to control esotropia.^1^,^5^,^6^

Binocular perception of depth is called stereopsis. It is the highest form of binocular vision and important for some professions such as pilots and ophthalmic surgeons who need high degree of hand-eye coordination.^7^ Maturation of stereopsis rapidly develops up to 8-18 months of age.^8^ Children with RAE may have satisfactory binocular vision due to occurrence of deviation after two years of age.^8^ On the other hand, many patients have abnormal binocular vision after perfect correction.^4^,^9^ The factors affecting stereoacuity were examined in many studies, but contradictory results were published.^10^ In this study, we sought to find out the factors affecting stereoacuity in patients with RAE.

## Methods

We retrospectively checked the charts of patients with RAE who visited our department between 1985-2011. The patients whose examinations were performed properly and data kept regularly, esotropia corrected to ortotropia with full hyperopic correction were included in the study. Exclusion criteria were mental retardation, neurological disease, systemic disorders, previous eye surgery, poor adaptation to stereoacuity tests, fewer than six months follow up time. Seventy patients who matched the criteria were included in our study.

Full ophthalmologic examination was performed on all of our patients. Children over five years of age had their refractive error evaluated by ARK-700 (Nidek Co. Ltd, Japan) auto refractometer, after using 2 drops of cyclopentolate 1%, and in patients under 5 years of age, retinoscopy was accomplished after administering two drops of atropine sulfate 0.5% for 3 days. Time between initiation age and age at first visit was assumed as duration of deviation. Visual acuity was measured with Snellen chart and E chart was used to test small children. Amblyopia was described as a difference of two or more Snellen lines between eyes and was treated with occlusion therapy. One diopter or more difference between eyes was defined as anisometropia. Full hyperopic correction was prescribed for treatment. Deviation at distance and near, with and without glasses was determined by Hirschberg test in small children and prism cover test in older patients. Binocular vision was examined using Titmus Test, Randot Test, Lang Test and fusion was examined using Bagolini lenses and Worth 4–dot Test.

Patients were classified into three groups at their final examination with the Titmus Test:

***Group 1:*** Stereoacuity score 40 second/arc
***Group 2:*** Stereoacuity score >40 second/arc (50-3000)
***Group 3:*** No binocular vision.

Multiple prognostic parameters that could affect stereoacuity were reviewed. Investigated parameters were; initiation age of RAE, consultation age, duration of deviation, refractive error, amblyopia, amblyopia treatment, anisometropia, visual acuity and angle of deviation both near (30 cm) and far (6 m).

Results were analyzed with SPSS 19.0 (Statistical Package for Scientific Studies for Windows, SPSS Inc., Chicago, IL). Kruskal–Wallis one-way analysis of variance and Pearson chi-square tests were used for statistical analysis. *P*<0.05 was assumed significant for all analysis. The procedures of the study were approved by the institutional review board of the hospital and adhered to the tenets of the Declaration of Helsinki.

## RESULTS

Seventy patients were included in the study. Thirty patients were female (42.9%), while forty patients were male (57.1%). Ten patients (14.3%) had family history of strabismus. Mean initiation age of RAE was 2.7±1.5 years (0-6 years), the mean age at first visit was 6.4±4.2 years (1-24 years). The mean follow up time was 7.3±4.4 years (6 months-19 years).

Seven patients (10%) had 40 second/arc, 48 patients (68.6%) had 50 to 3000 second/arc stereoacuity, 15 patients (21.4%) had no binocular vision and fusion was present in 13 of these patients. Among all patients, 55 patients (78.6%) had stereoacuity with the Titmus Test and Randot Test, 46 patients (65.7%) had stereoacuity with the Lang Test.

To investigate the prognostic factors that may affect stereoacuity score, comparisons were made between 3 groups. In total of 70 patients, 19 of them (27.1%) had amblyopia at final visit. There was statistically significant relation between amblyopia and stereoacuity (*P*=0.001). Comparison between initiation age of RAE and stereoacuity was not significant (*P*=0.69) and age at first visit was not related with the stereoacuity (*P*=0.21) Mean deviation for near was statistically higher in group 2 and 3 but no significant relation was observed between mean deviation for distance and stereoacuity (*P*=0.01, *P*=0.12). Duration of deviation was significantly different between 3 groups (*P*=0.01) Table-I. Visual acuity levels were higher in group 1 and 2. There was statistically significant relationship with stereoacuity and visual acuity (*P*=0.001, *P*=0.008). Mean spherical equivalent of the eyes were not statistically different between three groups. Grade of hyperopic refractive error was not significantly related with stereoacuity in either eye (*P*=0.07, *P*=0.21) (Table-II)

**Table-I:** Relationship between initiation age of deviation, age at first visit, duration of deviation, deviation for near and distance (without glasses), amblyopia, treatment of amblyopia, anisometropia and stereacuity.

	Group 1	Group 2	Group 3	p
Age at first visit	6.0±3.7	5.8±3.3	8.7±6.1	0.21**
İnitiation age of deviation	2.4±1.9	2.9±1.6	2.5±1.3	0.69**
Duration of deviation	2.0±1.4	2.4±3.5	5.0±4.1	0.01**
Deviation for near (Degree of arc)	+4.7±4.2	+6.9±6.3	+11.0±7.2	0.01**
Deviation for distance (Degree of arc)	+6.2 ±4.9	+6.5±4.5	+10.0±6.2	0.12**
Amblyopic patients	0.0 (0%)	9.0 (47.4 %)	10.0(52.6%)	0.001*
Amblyopia treatment	4.0 (13.8%)	24.0 (82.8%)	1.0 (3.4%)	0.01*
Anisometropia	1.0 (5.6%)	2.0 (66.7%)	5.0 (27.8%)	0.623*

* Pearson chi-square test

** Kruskal Wallis test, Mean± Standart deviation.

**Table-II:** Relationship between visual acuity, refractive error and stereacuity.

	Group 1	Group 2	Group 3	p*
	Right/Left	Right/Left	Right/Left	Right/Left
VA	1.0±0.0/1.0±0.0	0.9±0.0/0.9±0.0	0.8±0.2/0.8±0.2	0.001/0.008
RE	+3.4±1.3D/+3.8±1.3D	+4.9±1.8D/+5.0±1.7D	+5.4±2.5D/+5.5±2.5D	0.07/ 0.21

* Kruskal Wallis test,D: Dioptri Mean± Standart deviation, VA: Visual acuity, RE: Refractive error.

## DISCUSSION

RAE is induced by uncorrected hyperopia and is related with inadequate divergence, which causes an increase of convergence. Esotropia is treated with full correction of hyperopia by spectacles, contact lenses or refractive surgery.^11^-^13^ RAE usually leads to ambliyopia and damaged binocular vision, as treatment is often delayed treatment.^14^ In previous studies, relationship between stereopsis and visual acuity, refractive error, duration of deviation and amblyopia were reviewed. Some authors determined correlation between stereopsis and these parameters and reported stereopsis ratios between 50%-100% in patients with RAE.^4^,^15^ The relationship between stereopsis and initiation age of deviation, age at first visit, angle of deviation for near and distance, amblyopia treatment, anisometropia were relieved but results were contradictory. Berk at al reported 73.5% of the patients had fusion with Worth 4–dot Test.^16^ In our study, 82.8% of patients accomplished fusion. Uretmen et al found 480 seconds of arc or better was present in 21.8% of patients and 1.5% had 60 seconds of arc or better stereoacuity.^4^ Berk et al. demonstrated 22.5% had 1980-3000 seconds of arc, 21% had 200-800 seconds of arc, 24.2% had 40-100 seconds of arc stereopsis.^16^ Mulvihill et al reported 9.8% had 100-400 seconds of arc, 90.2% had 100 seconds of arc or better stereopsis and high stereoacuity were found to be related with late presentation of esotropia.^15^ Lambert and Lynn suggested 70% had gross stereopsis or no stereopsis, 18% had 120-480 seconds of arc, 12% had 15-60 seconds of arc stereopsis and high levels of steroeacuity were found in patients whose esotropia occurred at older age.^17^ Tomac reported 45% of patients had stereopsis.^18^ In the present study 10% of the patients had 40 seconds of arc, 68.6% of the patients had 50-3000 seconds of arc stereopsis and 21.4% of the patients had no stereopsis. We found similar results with other studies except Mulvihill’s study. We suggest that our low stereoacuity results may be due to amblyopia as a result the late presentation of our patients in seeking care.

Onset age of deviation may be clinically important for development of binocular vision. If binocular vision matures before onset of deviation, binocular functions will be better. Mean age of onset RAE noted by parents was 28.8-16.8 months.^19^ Mohney et al. defined mean age of onset 3.2 years.^20^ Mohan et al. reported mean age of RAE 2.78 years.^21^ Uretmen et al. determined onset age of RAE 31.2 months and did not find relationship between onset age of deviation and stereoacuity.^5^ On the other hand, Mulvihill et al. described patients whose deviation started at older to have high stereoacuity.^15^ Fawcett at al. reported patients with onset age of esotropia greater than 24 months had better stereoacuity.^8^ In our study, mean initiation age was not different between groups.

In present study, time between onset of deviation and initiation of treatment is thought to be important with regard to stereoacuity and initial patient presentation was quite late in our study. We found this period effective on binocular vision. In different studies there are conflicting results about the relation between duration of deviation and stereoacuity. In our study, mean duration of deviation was significantly different between groups. As a result, we suggest that period between onset of deviation and initiation of treatment is one of the most important determinants of stereoacuity.

Deviation is variable at RAE.^22^ The angle of esotropia is usually between 20-30 prism diopters (PD) and almost equal at near and far. Hutchinson et al found the mean deviation 18.6 PD both at distance and near.^23^ Mohney et al. also showed the angle of deviation at near 23.3 PD, at distance 17.3 PD.^20^ In our study, the mean angle of deviation at near was +11 degree of arc (19.3 PD), the mean angle of deviation at distance was +10 degree of arc (17.5 PD). We demonstrated that patients with higher angle of deviation at near had significantly lower stereoacuity. As a result, deviation at near is significantly related with stereoacuity and having clear near vision has an important role on development of stereopsis.

Hypermetropic errors of RAE patients were reported in many studies. Lai et al reported mean spherical equivalent +5.79±1.84D for both eyes.^24^ Gerling at al measured the mean cycloplegic refraction at initial visit +4.68D for right and +4.75D for left eyes.^25^ In our study, similar to the previous studies, we measured mean spherical equivalent at initial visit +4.1±1.7D in right and +4.2±1.8D in left eyes. Relation between grade of hyperopic error, anisometropia and stereoacuity were also investigated in previous studies. Uretmen et al reported mean spherical equivalent +4.75D in right, +5.0 D in left eye and they could not find relationship between stereoacuity and refractive error.^4^ Fawcett et al. reported that high hypermetropia was not risk factor for binocular vision in accommodative esotropia.^10^ We also did not observe any relation between them. On the other hand, we determined lowest visual acuity in group 3. As a result, we found significant relationship between visual acuity and stereoacuity. We consider that appropriate treatment as; full refractive correction and amblyopia treatment during the RAE is important for development of good stereopsis.

Patients with RAE have frequently amblyopia despite treatment and continuous examinations.^13^,^16^ Mulvihill et al. documented 61.2% of 103 patients had amblyopia and 15.5% of the patients were amblyopic at last examination.^15^ Berk et al. presented 59.2% of patients had amblyopia at the time of initial examination and 23% of patients were amblyopic at final examination.^16^ Lai et al. showed 82% of the patients were amblyopic at first visit and this rate decreased to 24% after treatment.^24^ Uretmen et al. reported 12.5% of the patients were amblyopic and they did not find any significant relation between amblyopia and stereoacuity.^4^ In the present study, amblyopic patients rate reduced from 77% to 27.2% between initial and final visit in accordance with previous studies. However, we found significant relation between amblyopia and poor stereoacuity. We determined that low visual acuity related with poor stereopsis and patients with low visual acuity had amblyopia too. But the incidence of amblyopia decreased dramatically after appropriate treatment. Hence, we might be able to make predictions for stereoacuity who had low visual acuity and amblyopia.

### Limitations of the study

First one is its retrospective design, second one is relatively small sample size, third one is retinoscopy and autorefractometer were used for some of the patients to evaluate the refractive error. The strength of our study is its long follow up period. We observed the long term effects of full refractive correction, amblyopia treatment and duration of deviation on stereopsis.

In conclusion, low visual acuity, higher angle of deviation at near, increased duration of deviation, presence of amblyopia and irregularity of amblyopia treatment were the prognostic factors for poor stereoacuity. To achieve better results, the importance of consulting an ophthalmologist at the beginning of symptoms should be emphasized to parents and children should be encouraged to wear their glasses full time.

## References

[1] Cho YA, Yi S, Kim S (2009). Clinical evaluation of cessation of hyperopia in 123 children with accommodative esotropia treated with glasses for best corrected vision. Acta Ophthalmologica.

[2] Rubin SE (2006). Bringing the management of accommodative esotropia into sharp focus. Am J Ophthalmol.

[3] Kim WJ, Kim MM (2014). Accommodative esotropia who needs spectacles for good ocular alignment after refractive shift below +2.00 Diopters. Korean J Ophthalmol.

[4] Uretmen O, Kose S, Oztaş Z, Egrılmez S (2007). Factors influencing stereoacuity in refractive accommodative esotropia. Can J Opthalmol**.

[5] DemirkilinçBiler E, Uretmen O, Köse S (2010). The effect of optical correction on refractive development in children with accommodative esotropia. JAAPOS.

[6] Somer D, Çınar FG, Duman S (2006). The accommodative element in accommodative esotropia. Am J Ophthalmol.

[7] Fielder AR, Moseley MJ (1996). Does stereopsis matter in humans? Eye.

[8] Fawcett S, Wang Y, Birch EE (2005). The critical period for suspceptibility of human stereopsis. Invest Ophthalmol Vis Sci.

[9] Birch EE, Wang J (2009). Stereoacuity outcomes following treatment of infantile and accommodative esotropia. Optom Vis Sci.

[10] Fawcett S, Birch EE (2003). Risk factors for abnormal binocular vision after successful alignment of accommodative esotropia. JAAPOS.

[11] Rossi S, Testa F, Santamaria C, Orrico A, Attanasio M, Simonelli F (2013). Photorefractive keratectomy on purely refractive accommodative esotropia. Seminars in Ophthalmology.

[12] Saeed AM, Abdrabbo MA (2011). LASIK as an alternative line to treat noncompliant esotropic children. Clin Ophthalmol.

[13] Shi M, Jiang H, Niu X, Dai H, Ye Y (2014). Hyperopic corneal refractive surgery in patients with accommodative esotropia and amblyopia. JAAPOS.

[14] Reddy AK, Freeman CH, Paysse EA, Coats DK (2009). A data-driven approach to the management of accommodative esotropia. Am J Ophthalmology.

[15] Mulvihill A, Maccann A, Flifcroft I, Keefe M (2000). Outcome in refractive accommodative esotropia. Br JOpthalmol.

[16] Berk T, Koçak N, Ellidokuz H (2004). Treatment outcomes in refractive accommodative esotropia. JAAPOS.

[17] Lambert SR, Lynn MJ (2006). Longitudinal changes in the spherical equivalent refractive error of children with accommodative esotropia. Br J Opthalmol.

[18] Tomac S (2002). Binocularity in refractive accommodative esotropia. J Pediatr Opthalmol Strabismus.

[19] Park K, Kım SM, OH SY (2011). The maximal tolerable reduction in hyperopic correction in patients with refractive accommodative esotropia. Am J Opthalmol.

[20] Mohney BG, Lilley CC, Green-Simms AE, Diehl NN (2011). The long-term follow-up of accommodative esotropia in a population based-cohort of children. Ophthalmology.

[21] Mohan K, Sharma A (2012). Development of refractive accommodative esotropia in children initially diagnosed with pseudoesotropia. J AAPOS.

[22] (2007-2008). American Academy of Ophthalmology. Pediatric Opthalmology and Strabismus.

[23] Hutchinson A, Serafino M, Nucci P (2010). Photorefractive keratectomy for the treatment of purely refractive accommodative esotropia: Six years experience. Br J Ophthalmol.

[24] Lai HC, Chen SH, Chen YF, Chiang YS, Yang ML (2004). Early predict the outcomes of refractive accommodative esotropia by initial presentations. Chang Gung Med J.

[25] Gerling A, Arnoldi K (2013). Single-Vision Lenses: A comparison of management of high AC/A esotropia and refractive esotropia. Strabismus.

